# Blood lipids and prostate cancer: a Mendelian randomization analysis

**DOI:** 10.1002/cam4.695

**Published:** 2016-03-19

**Authors:** Caroline J. Bull, Carolina Bonilla, Jeff M. P. Holly, Claire M. Perks, Neil Davies, Philip Haycock, Oriana Hoi Yun Yu, J. Brent Richards, Rosalind Eeles, Doug Easton, Zsofia Kote‐Jarai, Ali Amin Al Olama, Sara Benlloch, Kenneth Muir, Graham G. Giles, Robert J. MacInnis, Fredrik Wiklund, Henrik Gronberg, Christopher A. Haiman, Johanna Schleutker, Børge G. Nordestgaard, Ruth C. Travis, David Neal, Nora Pashayan, Kay‐Tee Khaw, Janet L. Stanford, William J. Blot, Stephen Thibodeau, Christiane Maier, Adam S. Kibel, Cezary Cybulski, Lisa Cannon‐Albright, Hermann Brenner, Jong Park, Radka Kaneva, Jyotsna Batra, Manuel R. Teixeira, Agnieszka Micheal, Hardev Pandha, George Davey Smith, Sarah J. Lewis, Richard M. Martin, The PRACTICAL consortium

**Affiliations:** ^1^ School of Social and Community MedicineUniversity of BristolBristolUnited Kingdom; ^2^ MRC/University of Bristol Integrative Epidemiology UnitUniversity of BristolBristolUnited Kingdom; ^3^ Integrative Cancer Epidemiology ProgrammeUniversity of BristolBristolUnited Kingdom; ^4^ IGFs and Metabolic Endocrinology GroupSchool of Clinical Sciences North BristolUniversity of BristolBristolBS10 5NBUnited Kingdom; ^5^ Department of MedicineDivision of Endocrinology, EpidemiologyBiostatistics and Occupational Health McGill UniversityMontrealQuebecCanada; ^6^ Department of Twin ResearchKing's College LondonLondonSE1 7EHUnited Kingdom; ^7^ The Institute of Cancer ResearchLondonSM2 5NGUnited Kingdom; ^8^ The Royal Marsden NHS Foundation TrustLondonSW3 6JJUnited Kingdom; ^9^ Centre for Cancer Genetic EpidemiologyDepartment of Public Health and Primary CareUniversity of CambridgeStrangeways LaboratoryWorts CausewayCambridgeUnited Kingdom; ^10^ Warwick Medical SchoolUniversity of WarwickCoventryCV4 7ALUnited Kingdom; ^11^ Institute of Population HealthUniversity of ManchesterManchesterM13 9PLUnited Kingdom; ^12^ Cancer Epidemiology CentreThe Cancer Council Victoria615 St Kilda RoadMelbourneVictoriaAustralia; ^13^ Centre for Epidemiology and BiostatisticsMelbourne School of Population and Global HealthThe University of MelbourneVictoriaAustralia; ^14^ Department of Medical Epidemiology and BiostatisticsKarolinska InstituteStockholmSweden; ^15^ Department of Preventive MedicineKeck School of MedicineUniversity of Southern California/Norris Comprehensive Cancer CenterLos AngelesCalifornia; ^16^ Department of Medical Biochemistry and GeneticsUniversity of Turku and Tyks Microbiology and GeneticsDepartment of Medical GeneticsTurku University HospitalTurkuFinland; ^17^ Institute of Biomedical Technology/BioMediTechUniversity of Tampere and FimLab LaboratoriesTampereFinland; ^18^ Department of Clinical BiochemistryHerlev and Gentofte HospitalCopenhagen University HospitalHerlev Ringvej 75HerlevDK‐2730Denmark; ^19^ Cancer EpidemiologyNuffield Department of Population HealthUniversity of OxfordOxfordUnited Kingdom; ^20^ Surgical Oncology (Uro‐Oncology: S4)University of CambridgeAddenbrooke's HospitalBox 279, Hills RoadCambridgeUnited Kingdom; ^21^ Cancer Research UK Cambridge Research InstituteLi Ka Shing CentreCambridgeUnited Kingdom; ^22^ Department of Applied Health ResearchUniversity College London1‐19 Torrington PlaceLondonWC1E 7HBUnited Kingdom; ^23^ Cambridge Institute of Public HealthUniversity of CambridgeForvie Site, Robinson WayCambridgeCB2 0SRUnited Kingdom; ^24^ Division of Public Health SciencesFred Hutchinson Cancer Research CenterSeattleWashington; ^25^ Department of EpidemiologySchool of Public HealthUniversity of WashingtonSeattleWashington; ^26^ International Epidemiology Institute1455 Research Blvd., Suite 550Rockville20850Maryland; ^27^ Mayo ClinicRochesterMinnesota; ^28^ Department of UrologyUniversity HospitalUlmGermany; ^29^ Institute of Human Genetics University HospitalUlmGermany; ^30^ Brigham and Women's Hospital/Dana‐Farber Cancer Institute45 Francis Street‐ ASB II‐3BostonMassachusetts02115; ^31^ Washington UniversitySt. LouisMissouri; ^32^ International Hereditary Cancer CenterDepartment of Genetics and PathologyPomeranian Medical UniversitySzczecinPoland; ^33^ Division of Genetic EpidemiologyDepartment of MedicineUniversity of Utah School of MedicineSalt Lake CityUtah; ^34^ Division of Clinical Epidemiology and Aging ResearchGerman Cancer Research Center (DKFZ)HeidelbergGermany; ^35^ Division of Preventive OncologyGerman Cancer Research Center (DKFZ) and National Center for Tumor Diseases (NCT)HeidelbergGermany; ^36^ German Cancer Consortium (DKTK)German Cancer Research Center (DKFZ)HeidelbergGermany; ^37^ Division of Cancer Prevention and ControlH. Lee Moffitt Cancer CenterMagnolia Dr.TampaFlorida12902; ^38^ Molecular Medicine Center and Department of Medical Chemistry and BiochemistryMedical University Sofia2 Zdrave StSofia1431Bulgaria; ^39^ Australian Prostate Cancer Research Centre‐QldInstitute of Health and Biomedical Innovation and School of Biomedical SciencesQueensland University of TechnologyBrisbaneAustralia; ^40^ Department of GeneticsPortuguese Oncology InstitutePortoPortugal; ^41^ Biomedical Sciences Institute (ICBAS)Porto UniversityPortoPortugal; ^42^ The University of SurreySurreyGU2 7XHUnited Kingdom; ^43^ National Institute for Health ResearchBristol Nutrition Biomedical Research UnitBristolUnited Kingdom

**Keywords:** Cholesterol, Mendelian randomization, prostate cancer, statins

## Abstract

Genetic risk scores were used as unconfounded instruments for specific lipid traits (Mendelian randomization) to assess whether circulating lipids causally influence prostate cancer risk. Data from 22,249 prostate cancer cases and 22,133 controls from 22 studies within the international PRACTICAL consortium were analyzed. Allele scores based on single nucleotide polymorphisms (SNPs) previously reported to be uniquely associated with each of low‐density lipoprotein (LDL), high‐density lipoprotein (HDL), and triglyceride (TG) levels, were first validated in an independent dataset, and then entered into logistic regression models to estimate the presence (and direction) of any causal effect of each lipid trait on prostate cancer risk. There was weak evidence for an association between the LDL genetic score and cancer grade: the odds ratio (OR) per genetically instrumented standard deviation (SD) in LDL, comparing high‐ (≥7 Gleason score) versus low‐grade (<7 Gleason score) cancers was 1.50 (95% CI: 0.92, 2.46; *P* = 0.11). A genetically instrumented SD increase in TGs was weakly associated with stage: the OR for advanced versus localized cancer per unit increase in genetic risk score was 1.68 (95% CI: 0.95, 3.00; *P* = 0.08). The rs12916‐T variant in 3‐hydroxy‐3‐methylglutaryl‐CoA reductase (HMGCR) was inversely associated with prostate cancer (OR: 0.97; 95% CI: 0.94, 1.00; *P* = 0.03). In conclusion, circulating lipids, instrumented by our genetic risk scores, did not appear to alter prostate cancer risk. We found weak evidence that higher LDL and TG levels increase aggressive prostate cancer risk, and that a variant in HMGCR (that mimics the LDL lowering effect of statin drugs) reduces risk. However, inferences are limited by sample size and evidence of pleiotropy.

## Introduction

Prostate cancer is the most prevalent male cancer in Europe and a major cause of cancer‐related deaths 1. Lifestyle factors and related intermediate phenotypes have been associated with prostate cancer development and progression in epidemiological studies, including a positive association between circulating cholesterol levels and prostate cancer 2, 3, 4. However, conclusions are conflicting and it is not clear whether these findings reflect causality or are the product of confounding by common causes of both cholesterol levels and prostate cancer (e.g., aspects of diet), bias, or reverse causality (the cancer causing altered cholesterol metabolism) 5. As serum cholesterol levels can be modified by lifestyle changes 6 and statin therapy 7, clarifying the causality of this association could inform the development of prevention interventions for prostate cancer.

Statins lower cholesterol levels by inhibiting HMG‐CoA reductase, the rate‐limiting enzyme in cholesterol synthesis. A meta‐analysis of 27 observational studies concluded that statin therapy reduced prostate cancer by 7% (RR 0.93; 95% CI: 0.87, 0.99; *P* = 0.03) 8, but a separate meta‐analysis of four randomized trials showed minimal evidence of any association (RR 1.08; 95% CI: 0.91, 1.30; *P* = 0.38) 9. Both analyses revealed considerable heterogeneity between the included studies (*I* ^2^ values >70%) and, as prostate cancer was assessed as a secondary outcome in the trials, misclassification of outcome could have biased the results. As yet, evidence regarding statin therapy for prostate cancer is controversial 10.

**Figure 1:**
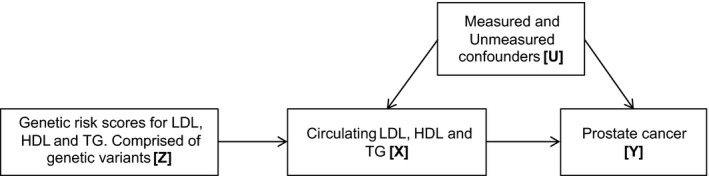
Mendelian randomization. Using genetic variants as instrumental variables to establish whether an exposure is causally related to cancer. An instrumental variable (genetic variation) [Z] acts as a proxy for environmental exposure [X], postulated to influence cancer [Y]. Z is independent of measured or unmeasured confounders [U]. Z only influences Y if X →Y is causal.

Mendelian randomization uses genetic variants robustly associated with traits of interest (in this case, circulating low‐density lipoprotein [LDL], high‐density lipoprotein [HDL], and triglyceride [TG] levels) as instrumental variables to make inferences about whether associations between exposures and disease are likely to be causal 11. The principle of Mendelian randomization is that analysis of groups defined by common genetic variants is analogous to that of an intention‐to‐treat analysis in a randomized controlled trial, based on Mendel's laws of segregation and independent assortment. Using genetic variants as “instrumental variables” to proxy modifiable exposures should be unconfounded by environmental factors, represent life‐long exposure, and not be subject to reverse causality with respect to the phenotype proxied by the genotype. A genetic score using several single nucleotide polymorphisms (SNPs) in combination can be constructed to represent the additive effect of multiple gene variants to explain more of the variance in the risk factor of interest, avoiding weak instrument bias, and increasing power 12. Genetic scores using multiple gene variants for lipid traits have been used previously to investigate associations between blood lipids and vascular disease 13, 14, 15. If the instrumental variable assumptions hold, as shown in Figure 1, then a test of the association between the instrument and the outcome is a test of the presence of a causal effect of the intermediate on the outcome 16.

Here we test for the possible presence of a causal effect of lipid fractions (LDL, HDL, TG) on prostate cancer using genetic variants for these traits combined in genetic risk scores using the principle of Mendelian randomization 11. As far as we know, this study is the first to employ Mendelian randomization to investigate the association between circulating lipids and prostate cancer risk.

## Methods

Genetic risk scores as instruments for circulating lipid fractions were developed using SNPs previously identified by genome‐wide association studies (GWAS). These scores were then applied to 22,249 prostate cancer cases and 22,133 controls within the international PRACTICAL consortium 17 for whom genetic data were available.

### Study populations

#### PRACTICAL consortium (prostate cancer association group to investigate cancer‐associated alterations in the genome)

We investigated the association between genetic risk scores for lipid traits and prostate cancer risk in an individual participant meta‐analysis of men in 22 studies of the international PRACTICAL consortium (Table 1). Fifteen of these studies were based in Europe, five in North America, and two in Australia. Genotypic information was available for 44,382 participants (22,249 prostate cancer cases and 22,133 controls). Subjects were predominantly of European descent (mean across studies = 99%). All studies met the appropriate ethical criteria for each country in accordance with the principles embodied in the Declaration of Helsinki. Further details are available from the consortium website (http://www.practical.ccge.medschl.cam.ac.uk).

**Table 1:** Summary of 22 PRACTICAL case–control studies, *n* = 44,382 men

Study	Acronym	Country	n		Mean		%			
			Controls	Cases	Age at diagnosis (years)	PSA at diagnosis (ng/mL)	European ethnicity	Family history of prostate cancer	High grade^a^	Advanced cancer^a^
Cancer of the Prostate in Sweden	CAPS	Sweden	664	1153	66.7	79.6	100	11.9	50.0	30.3
Copenhagen Prostate Cancer Study 1	CPCS1	Denmark	2771	848	59.5	48.0	100	8.2	71.2	—
Copenhagen Prostate Cancer Study 2	CPCS2	Denmark	1009	265	58.0	36.0	100	14.7	52.2	—
European Prospective Investigation Into Cancer and Nutrition (BPC3)	EPIC	Europe	1079	722	61.7	—	100	—	27.9	4.0
Epidemiological investigations of the chances of preventing, recognizing early and optimally treating chronic diseases in an elderly population	ESTHER	Germany	318	313	65.4	58.7	100	6.4	48.0	27.6
Fred Hutchinson Cancer Research Centre	FHCRC	USA	729	761	59.7	16.1	91.4	16.2	41.7	20.2
Portuguese Oncology Institute, Porto	IPO‐Porto	Portugal	66	183	54.0	8.3	100	20.0	84.2	13.1
Mayo Clinic	MAYO	USA	488	767	65.3	15.5	100	23.3	55.3	45.5
Melbourne Collaborative Cohort Study	MCCS	Australia	1170	1698	56.0	19.4	99.8	29.6	53.1	14.5
Multiethnic Cohort Study (BPC3)	MEC	USA	829	819	70.0	—	100	10.7	—	12.5
The Moffitt Group	MOFFITT	USA	100	414	64.5	6.5	89.4	20.1	43.1	3.5
Prostate Cancer study Medical University Sofia	PCMUS	Bulgaria	140	151	68.2	23.1	100	4.1	59.6	46.7
The Poland Group	Poland	Poland	359	438	65.5	40.2	100	6.9	32.8	37.1
Prostate Project Foundation–Postgraduate Medical School, Surrey	PPF‐UNIS	UK	188	245	67.9	19.3	99.6	25.2	45.5	28.2
Prostate testing for cancer and Treatment	ProtecT	UK	1474	1563	61.2	6.3	99.7	7.0	29.9	11.4
Retrospective Queensland Study (QLD) and the Prostate Cancer Supportive Care and Patient Outcomes Project (ProsCan)	QLD	Australia	87	186	64.3	6.7	100	25.9	83.1	—
Study of Epidemiology and Risk factors in Cancer Heredity	SEARCH	UK	1244	1371	58.9	53.2	99.9	16.2	56.8	17.8
Stockholm 1	STHMI	Sweden	2224	2006	66.7	—	100	17.1	45.5	14.4
Finnish Genetic Predisposition to Prostate Cancer Study	TAMPERE	Finland	2413	2754	68.2	69.1	100	—	43.7	21.4
U.K. Genetic Prostate Cancer Study and The Prostate Cancer Research Foundation Study	UKGPCS	UK	4182	4549	61.1	46.9	100	24.7	50.5	36.0
Institut fuer Humangengetik Ulm	ULM	Germany	354	603	62.4	19.1	100	33.4	51.3	40.5
UTAH Study	UTAH	USA	245	440	64.0	—	100	33.1	—	17.2

Missing data were excluded from each analysis.

—, no data available.

a Advanced cancer (T3 to T4 or SEER staging regional or distant), high grade (Gleason ≥7), low grade (Gleason ≤6).

Data concerning cancer grade and stage were collected by each study. Cancers were categorized as low grade (Gleason score ≤6) or high grade (Gleason score ≥7), and localized (T1 or T2 on TNM staging, or if not available, “localized” on SEER staging) or advanced (T3 or T4 on TNM staging, or if not available, “regional” or “distant” on SEER staging). Data were not available for grade in two (MEC/UTAH), or stage in three (CPCS1/CPCS2/QLD), of the PRACTICAL studies, respectively.

#### GWAS identification of lipid SNPs

SNPs associated with the lipid traits under investigation were identified by a review of the current published literature in which papers concerning lipid SNPs were identified by entering appropriate search terms into the web of science database (“Lipid,” “SNP,” “GWAS”). We selected SNPs that were exclusively associated, at genome‐wide significance, with each lipid trait of interest. The majority of SNPs were taken from two landmark lipid GWAS 18, 19, the latter being the largest genetic association study of blood lipid levels to be conducted to date (188,577 individuals). For completeness, a number of SNPs from other publications were also included in the analysis 14, 20. The EPIC‐Norfolk cohort was used by the referenced GWASs to identify SNPs associated with lipid traits; we therefore excluded this study from our analysis to reduce the chance of under‐estimating the causal effect of the lipids on prostate cancer.

#### Genotyping data

PRACTICAL samples were genotyped using an Illumina Custom Infinium genotyping array (iCOGS) consisting of 211,155 SNPs designed for the Collaborative Oncological Gene–Environment Study (COGS) (details of which may be found on their website http://www.cogseu.org). The array was specifically designed for the evaluation of genetic variants in breast, ovarian, and prostate cancer. As genotypic information was not available for all SNPs in the genetic risk scores, we also used SNPs that had been imputed using IMPUTE2 software 21. As a sensitivity analysis, allele frequencies and ORs for prostate cancer outcomes using imputed and genotyped data were compared by cross tabulation (Table S1). As results were similar, imputed data were used in all subsequent analyses. All SNPs with an indication of poor imputation quality were removed (*r* ^2^ hat<0.30), as were those with a minor allele frequency of <1%, a call rate of <95%, or those that violated the Hardy–Weinberg equilibrium (*P* < 0.05).

#### Gene variants used to create the genetic risk scores

Genetic risk scores were used as instruments to proxy exposure to circulating blood lipids in a Mendelian randomization framework 22. This analysis assumes that the genetic risk scores used in the analysis influence prostate cancer risk only via their ability to alter the specific lipid trait that they are acting as proxies for (e.g., LDL, Fig. 1). If other biochemical processes or traits are associated with the genetic risk scores (i.e., horizontal pleiotropy is present, where pathways from lipid‐associated SNPs to disease are involved that are independent of lipids, see Box 1) and also directly alter prostate cancer risk, this violates a major assumption of Mendelian randomization by introducing genetic confounding. Therefore, only SNPs exclusively associated at genome‐wide significance (*P* < 5 × 10^−8^) with one lipid trait (either LDL or HDL or TGs, but not more than 1) were chosen for each genetic risk scores to make them as specific to one trait as possible (as in Holmes et al. 15) (Table S2), minimizing the possibility of pleiotropic effects. Of the 118 SNPs identified as associated with only one lipid trait, 62 were either genotyped or imputed in the PRACTICAL consortium and considered eligible for inclusion in the genetic risk scores based on current biological knowledge. SNPs selected for the genetic risk scores were tested for evidence of linkage disequilibrium (LD) using the SNAP pairwise online tool (http://www.broadinstitute.org/mpg/snap/ldsearchpw.php), an open access resource which uses pairwise LD data based on phased genotype data from the International HapMap project. A threshold *r* ^2^ value of ≥0.85 was used to indicate LD; where SNPs were in LD, the SNP with the largest effect on the lipid trait was selected for the genetic risk scores. SNP genotypes were coded as 0, 1, or 2 depending on exposure to the risk allele. Dosage values (ranging from 0 to 2) were generated for imputed SNPs. LDL, HDL, and TG scores were composed of 11, 36, and 15 SNPs, respectively. Genetic risk scores were created by summing the number of “risk” alleles that each of the 22,249 prostate cancer cases and 22,133 controls were exposed to, such that the greater the number of “risk” alleles a man had, the higher the score. “Risk” alleles were those that were positively related to serum LDL or TG, or negatively related to serum HDL. Published effect sizes (the effect of the risk allele on the trait in SD) were applied to each SNP and summated to give a “weighted” genetic risk score for each trait 23, for each individual man in each study, so that when estimating the effect of a unit increase in the genetic risk score directly translated to the effect of a SD change in the trait upon the outcome.

Box 1 **Pleiotropy**

Vertical pleiotropy: A genetic locus is linked to a cascade of events. This is not generally a problem for Mendelian randomization studies.

Horizontal pleiotropy: A genetic locus is related to multiple phenotypes. This violates Mendelian randomization assumptions.

In a subsidiary analysis we examined the association of a SNP in 3‐hydroxy‐3‐methylglutaryl‐CoA reductase (HMGCR) (rs12916) in relation to prostate cancer outcomes. rs12916‐T has been used previously to mimic statin intervention in order to estimate the causal association of statin use with type 2 diabetes and adiposity measures 24. ORs for prostate cancer outcomes were reported per rs12916‐T allele.

#### Validation of genetic risk scores and investigation of potential pleiotropy in the ALSPAC cohort

The Avon Longitudinal Study of Parents and Children (ALSPAC) is a birth cohort established to investigate environmental and genetic factors in health and development 25. ALSPAC data were used to validate the genetic risk scores as instruments for circulating lipid exposure using linear regression. Ten of the 11 SNPs in the LDL score could be included in score validation analyses using ALSPAC data (due to poor imputation quality in ALSPAC, rs1801689 could not be included in the LDL score validation). Thirty‐five of the 36 HDL genetic risk score SNPs could be validated in ALSPAC (rs1084651 was poorly imputed) and 14 of the 15 TG genetic risk score SNPs were available for validation in the ALSPAC cohort (rs11649653 was poorly imputed).

### Statistical analysis

The genetic risk scores were entered into logistic regression models to estimate the effect per genetically instrumented SD increase in the lipid trait on prostate cancer outcomes.

Outcomes investigated were: all prostate cancer (case vs. control status), grade (high [≥7] versus low [≤6] Gleason score), and stage (advanced versus localized TNM or SEER staging). We conducted the analyses within each of the individual studies that make up the PRACTICAL consortium and then combined the results into a summary odds ratio (OR) for each outcome per unit increase in the genetic risk scores by fixed effect meta‐analysis using the “metan” command in Stata v.13 26. To test that the instruments (genetic risk scores) were not associated with confounders, we investigated whether each genetic risk score was associated with available covariables that could be potential confounding factors (age, diagnostic prostate specific antigen (PSA) levels, and family history of prostate cancer). To account for potential confounding by population stratification, we adjusted for the top eight principle components (variables concerning the population's genetic architecture). All analyses were performed in Stata v.13 (Stata Corp LP, 2013, College Station, TX).

## Results

The genetic risk scores were validated in ALSPAC participants at the age of 7 years (Table 2). The genetic risk score for LDL explained 0.3% of the variability in circulating LDL and not only was most strongly associated with LDL (linear regression coefficient; 0.56 mmol/L LDL; *F* stat: 14.11; *P* = 2 × 10^−4^), but was also weakly associated with HDL (linear regression coefficient; 0.19 mmol/L HDL; *F* stat: 5.32; *P* = 0.02). The HDL genetic risk score was the strongest instrument, explaining 0.9% of variability in circulating HDL and was associated exclusively with HDL (linear regression coefficient; −0.25 mmol/L HDL; *F* stat: 37.60; *P* = 9.5 × 10^−10^). The TG genetic risk score was most strongly associated with TG and explained 0.2% of the variability in circulating TG (linear regression coefficient; 0.35 mmol/L lnTG; *F* stat: 9.76; *P* = 2 × 10^−3^); however, it was also associated with HDL (0.15% variability explained, linear regression coefficient; −0.19 mmol/L HDL; *F* stat: 6.28; *P* = 0.01). Associations of the genetic risk scores with each lipid trait remained unaltered following stratification of participants by sex (data available on request).

**Table 2:** Weighted genetic risk score validation in ALSPAC (*N *= 4081)

Genetic risk score	Change in trait levels (mmol/L) per unit score^a^	95% CI	*P* value	*r* ^2^ (%)	*F*
LDL (10 SNPs)					
LDL	0.56	0.27, 0.86	2 × 10^−4^	0.34	14.11
HDL	0.19	0.03, 0.35	0.021	0.13	5.32
lnTG	−0.18	−0.41, 0.06	0.141	0.05	2.17
HDL (35 SNPs)					
HDL	−0.25	−0.32, −0.17	9.50 × 10^−10^	0.91	37.60
LDL	−0.12	−0.25, 0.04	0.16	0.05	1.97
lnTG	0.05	−0.06, 0.17	0.36	0.02	0.36
TG (14 SNPs)					
lnTG	0.35	0.13, 0.57	0.002	0.24	9.76
LDL	0.12	−0.16, 0.39	0.40	0.02	0.70
HDL	−0.19	−0.34, −0.04	0.01	0.15	6.28

a TG levels have been natural log transformed.

Individual data from 22,249 case and 22,133 control men in 22 PRACTICAL studies were included in our analysis (Table 1). The percentage of high‐grade cancers reported varied between studies (27.9–84.2%), as did the proportion of advanced stage cancers (3.5–46.7%). There was little evidence to support an association between genetic risk scores and PSA at recruitment. Family history of prostate cancer was weakly, and imprecisely, associated with the LDL (OR: 0.50, 95% CI: 0.21, 1.19; *P* = 0.12) and HDL (OR: 1.62, 95% CI: 1.02, 2.58; *P* = 0.04) genetic risk scores. Four of the top eight principal components were associated with at least one of the genetic risk scores. Age at interview was not associated with the genetic risk scores, with the exception of the TG score (linear regression coefficient: −2.76, 95% CI: −5.52, −0.0003; *P* = 0.05) (Table 3).

**Table 3:** Association genetic risk scores with potential confounding variables in 22,133 PRACTICAL control men

Variable	*n*	LDL	HDL	TG
Change in variable per unit increase genetic risk score^a^ (95% CI), *P* value				
Principle component 1	22,133	−0.20 (−0.45, 0.04), 0.10	−0.08 (−0.29, 0.14), 0.47	−0.17 (−0.51, 0.16), 0.30
Principle component 2	22,133	0.80 (0.43, 1.17), 2 × 10^−4^	−0.06 (−0.19, 0.07), 0.35	0.70 (0.24, 1.15), 4 × 10^−3^
Principle component 3	22,133	−0.54 (−0.81, −0.27), 4 × 10^−4^	0.01 (−0.10, 0.12), 0.82	−0.36 (−0.60, −0.13), 4 × 10^−3^
Principle component 4	22,133	0.28 (−0.19, 0.74), 0.23	0.06 (−0.15, 0.28), 0.54	−0.49 (−0.82, −0.16), 0.01
Principle component 5	22,133	0.35 (−0.18, 0.87), 0.18	−0.10 (−0.29, 0.09), 0.29	0.21 (−0.20, 0.63), 0.30
Principle component 6	22,133	−0.56 (−1.00, −0.15), 0.01	0.11 (−0.06, 0.28), 0.19	0.19 (−0.19, 0.57), 0.32
Principle component 7	22,133	−0.08 (−0.39, 0.23), 0.61	0.23 (−0.06, 0.53), 0.11	0.03 (−0.39, 0.44), 0.90
Principle component 8	22,133	0.28 (−0.19, 0.75), 0.22	0.03 (−0.18, 0.25), 0.75	0.09 (−0.23, 0.41), 0.58
PSA (ng/mL)	5012	−0.08 (−1.79, 1.63), 0.86	−0.16 (−0.56, 0.23), 0.22	0.17 (−0.42, 0.76), 0.35
Age (years)	18,962	−0.68 (−3.80, 2.43), 0.65	−0.33 (−2.24, 1.58), 0.73	−2.76 (−5.52. −0.0003), 0.05
OR family history per unit increase in genetic risk score (95% CI), *P* value				
Family history^b^	10,955	0.50 (0.21, 1.19), 0.12	1.62 (1.02, 2.58), 0.04	0.60 (0.25, 1.42), 0.24

Studies with more than 20% missing data were excluded from each analysis. Linear regression models take clustering by substudy into account.

LDL, low‐density lipoprotein; HDL, high‐density lipoprotein; TG, triglyceride; OR, odds ratio.

a Higher LDL/TG scores reflect increasing circulating LDL/TG, higher HDL scores reflect lower circulating HDL.

b Family history of prostate cancer (in father or brother): compares Yes versus No (logistic regression).

**Figure 2:**
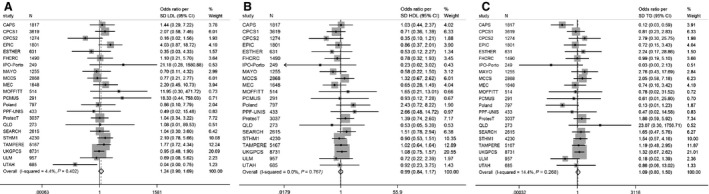
Meta‐analysis OR prostate cancer per unit increase in genetic risk scores (SD trait). (A) Low‐density lipoprotein (LDL): OR 1.24 (95% CI: 0.90, 1.69),*P* = 0.18. (B) High‐density lipoprotein (HDL): OR 0.99 (95% CI: 0.84, 1.17), *P* = 0.90. (C) Triglyceride (TG): 1.09 (95% CI: 0.80, 1.50), *P* = 0.57. Cases: 22,249; controls: 22,133. Adjusted for top eight principle components.

The pooled ORs for overall prostate cancer risk by lipid trait, estimated in instrumental variable analysis using the genetic risk score, were 1.24 (95% CI: 0.90, 1.69; *P* = 0.18; *I* ^2^ = 4.4%), 0.99 (95% CI: 0.84, 1.17; *P* = 0.90; *I* ^2^ = 0%), and 1.09 (95% CI: 0.80, 1.50; *P* = 0.57; *I* ^2^ = 14.4%) per genetically instrumented SD increase in LDL, HDL, and TG, respectively (Fig. 2).

The ORs for prostate cancer outcomes stratified by grade and stage are summarized in Table 4. There was weak evidence to suggest an effect for a genetically instrumented SD increase in LDL between high‐ versus low‐grade cancer cases (OR: 1.50; 95% CI: 0.92, 2.46; *P* = 0.11). There was little evidence to suggest an association between the HDL and TG genetic risk scores and prostate cancer grade: When high‐ and low‐grade cancers were compared, a genetically instrumented SD decrease in HDL gave an OR of 1.03 (95% CI: 0.79, 1.34; *P* = 0.82), and a genetically instrumented SD increase in TG gave an OR of 0.93 (95% CI: 0.57, 1.52; *P* = 0.77). For cancer stage, a genetically instrumented SD increase in LDL gave an OR for advanced versus localized cancers of 0.91 (95% CI: 0.51, 1.64; *P* = 0.77). A genetically instrumented SD decrease in HDL gave an OR for advanced versus localized cancers of 1.02 (95% CI: 0.74, 1.39; *P* = 0.92). The OR for advanced versus localized prostate cancer per SD genetically instrumented increase in TG was 1.68 (95% CI: 0.95, 3.00; *P* = 0.08).

**Table 4:** Case‐only analysis: weighted genetic risk scores and prostate cancer stage and grade (PRACTICAL consortium)

Outcome	Localized/low grade (*n*)	Advanced/high grade (*n*)	OR^a^	95% CI	*P* value
LDL score					
Advanced versus localized	13,707	4301	0.91	0.51, 1.64	0.77
High grade versus low grade	9237	8515	1.50	0.92, 2.46	0.11
HDL score					
Advanced versus localized	13,707	4301	1.02	0.74, 1.39	0.92
High grade versus low grade	9237	8515	1.03	0.79, 1.34	0.82
TG score					
Advanced versus localized	13,707	4301	1.68	0.95, 3.00	0.08
High grade versus low grade	9237	8515	0.93	0.57, 1.52	0.77

Advanced (T3 to T4 or SEER staging regional or distant), localized (T1 to T2 or SEER staging localized), high grade (Gleason ≥7), low grade (Gleason ≤6).

LDL, low‐density lipoprotein; HDL, high‐density lipoprotein; TG, triglyceride; OR, odds ratio.

a Per unit increase in genetic risk score (SD trait), adjusted for top eight principal components (higher LDL/TG scores reflect increasing circulating LDL/TG, higher HDL scores reflect lower circulating HDL).

EPIC‐Norfolk was excluded from the analysis, as the cohort was included in the referenced GWAS discovery panels. As a sensitivity analysis, we included EPIC‐Norfolk in the meta‐analysis. The results for EPIC‐Norfolk were in agreement with the combined estimates for the odds of prostate cancer by the genetic risk scores (Fig. S1). As some associations were present between the genetic risk scores and family history or age at diagnosis (Table 3), we performed a sensitivity analysis adjusting for these variables. The results were unaltered following adjustment for age with the TG score and for family history with the LDL and HDL scores (data available on request).

**Figure 3:**
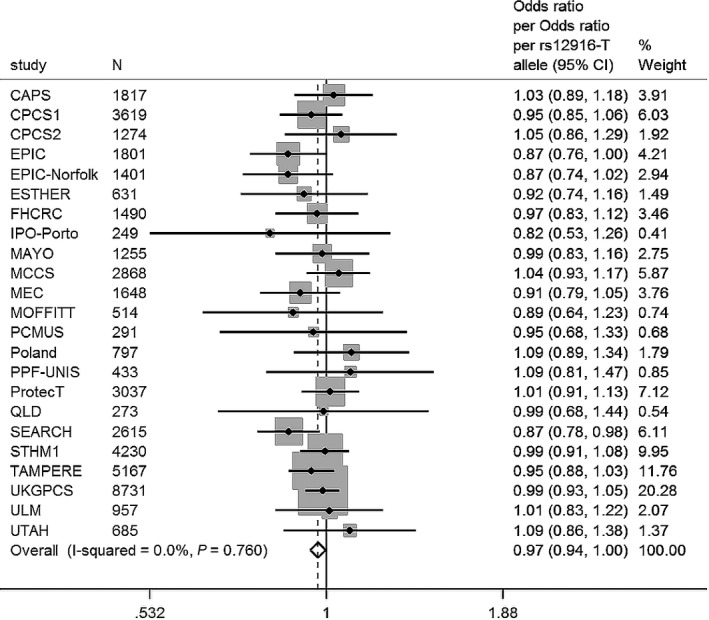
Cases versus controls. Meta‐analysis OR prostate cancer per rs12916‐T allele. OR 0.97 (95% CI: 0.94, 1.00), *P* = 0.03. Cases: 22,733; controls: 23,050. Adjusted for top eight principle components.

The rs12916‐T variant in HMGCR was weakly associated with a decreased risk of prostate cancer overall (OR: 0.97; 95% CI: 0.94, 1.00; *P* = 0.03; *I* ^2^ = 0%) (Fig. 3). There was little evidence of an association with prostate cancer stage (OR, advanced vs. localized: 0.97; 95% CI: 0.92, 1.02; *P* = 0.26) or grade (OR, high vs. low: 1.03; 95% CI: 0.98, 1.07; *P* = 0.21) (Table 5).

**Table 5:** Stratified analysis by cancer stage and grade. OR per rs12916‐T allele. Adjusted for top eight principle components

Outcome	Localized/low grade (*n*)	Advanced/high grade (*n*)	OR	95% CI	*P* value
rs12916‐T allele					
Advanced versus localized	13,707	4301	0.97	0.92, 1.02	0.26
High grade versus low grade	10,038	8543	1.03	0.98, 1.07	0.21

Advanced (T3 to T4 or SEER staging regional or distant), localized (T1 to T2 or SEER staging localized), high grade (Gleason ≥7), low grade (Gleason ≤6).

OR, odds ratio.

## Discussion

We did not find evidence to suggest an association between circulating LDL, HDL, or TG (proxied by genetic risk scores) and overall prostate cancer. Although power to investigate advanced/high‐grade cancer is limited (as evidenced by wide confidence intervals), our results may indicate a potential role for LDL and TG in prostate cancer progression. These findings are clinically important, as they highlight a distinction between indolent disease and more aggressive cancers. Variation in *HMGCR* has been exploited previously to demonstrate the implications of statin treatment on type 2 diabetes and bodyweight 24. Our results from an analysis using a variant in *HMGCR* (rs12916‐T) to proxy statin intervention suggest that statins may hold potential in prostate cancer prevention, but our inference is tentative and requires further investigation in larger sample sizes. Associations observed between the LDL and HDL genetic risk scores and family history findings may be an artifact of multiple testing. However, these findings warrant further investigation with larger numbers to increase the precision of the point estimates.

Meta‐analyses of observational studies and randomized controlled trials present heterogeneous findings for associations of cholesterol with prostate cancer, making it difficult to conclude whether cholesterol plays a role in prostate cancer. The most recent and comprehensive assessment of observational data did not show any association between HDL and LDL and prostate cancer, but could not draw definitive conclusions on high‐grade prostate cancer due to limited data 5. Our Mendelian randomization approach has several advantages over conventional observational epidemiology: it eliminates the problem of reverse causality, as prostate cancer status cannot alter one's germline genetic makeup; genetic risk scores represent an individual's exposure to lipid traits over their lifetime, reducing biological and technical sources of measurement error that arise from one‐off blood sampling at one point in a person's life; and confounding by behavioral, lifestyle, and other related intermediate traits should be minimized as individuals are effectively randomly allocated to a low or high level of exposure based on their genotype, randomly generated at conception (Mendel's second law of independent assortment) 27. However, Mendelian randomization is susceptible to genetic confounding if the SNPs used as instruments for the trait of interest have effects on other phenotypes besides the specific lipid of interest, and it is these other phenotypes which lead to prostate cancer (horizontal pleiotropy) 16. There is also potential for confounding due to population stratification; however, as we have adjusted for principle components in our regression models, this should be minimized. We cannot be sure that the LDL score is exclusively associated with LDL as validation of the score in ALSPAC revealed possible pleiotropy with circulating HDL. However, the HDL score which is strongly associated with HDL, and not LDL or TG in ALSPAC, was not associated with prostate cancer; therefore, the weak association noted between the LDL score and high‐grade prostate cancer is likely an LDL, not an HDL effect. As *F* statistics for the genetic risk scores relate to ALSPAC and not PRACTICAL (the dataset for the outcome), conventional thresholds, such as *F* > 10 are not relevant for this study. The dataset for the outcome is far larger than the dataset in which we tested the genetic risk score–trait association; therefore, it may be that the genetic risk scores are in fact stronger instruments for circulating lipid traits in PRACTICAL than we estimated.

A recent meta‐analysis of 14 prospective studies reported that blood LDL and HDL were not associated with either overall prostate cancer or high‐grade cancers; however, subgroup analysis was only performed on a limited number of studies and it is likely that the study was underpowered to detect any effect of these traits on cancers stratified by stage and grade 5. Our findings suggestive of a possible role for LDL in high‐grade prostate cancer and for TG in advanced stage prostate cancer are supported by Andreassen et al., who found evidence of an association between these traits and prostate cancer with the use of an alternative genetic epidemiologic method 28; however, this analysis was also conducted with data obtained from the PRACTICAL consortium. Using conjunction false discovery rate analysis, they were able to combine summary statistics from GWAS for the identification of genetic overlap between the two phenotypes (blood lipids and prostate cancer). Similarly, they found no pleiotropic enrichment for HDL in prostate cancer.

A meta‐analysis of 27 observational studies reported a 7% relative reduction in total prostate cancer risk with statin therapy 8. Our results for the rs12916‐T SNP and prostate cancer outcomes support similar conclusions; however, it is possible that the observed effects are via the increase in type 2 diabetes phenotypes associated with this allele 24, as an inverse association between type 2 diabetes and prostate cancer has been reported 29.

Cholesterol is thought to have multiple procancer effects at the cellular level: it is involved in cellular proliferation, inflammation, membrane organization, and steroidogenesis. Our findings are supported by preclinical work showing increasing concentrations of LDL support the proliferation of prostate cancer cell lines, but not normal epithelial cells, suggesting cholesterol metabolism is reprogrammed in prostate cancer 30. Furthermore, as signal transduction proteins are located in cholesterol‐rich membranes, it makes sense that oncogenic signaling might be regulated in a cholesterol‐sensitive manner 31. As statins are largely retained in liver, any effects on prostate cancer are not likely directly on the tissue, but via their ability to reduce circulating cholesterol 32. Statins have also been associated with similar cancer phenotypes in cellular and animal models, indicating that the effects of statins observed in populations are likely due to on‐target effects of statins (i.e., their ability to reduce circulating cholesterol via HMG‐CoA reductase inhibition) rather than off‐target effects, although these have been reported 33, 34, 35. Further research on this subject is needed, as previous work has found that statins differentially affect prostate cancer depending on their subtype 36 and the cancer phenotype 32.

A large number of genes robustly associated with serum lipids were used in the generation of genetic risk scores and their association (at genome‐wide significance level) with just one of the lipid traits may mitigate against results being due to pleiotropy with other lipids. However, key SNPs likely regulate multiple traits, so would therefore have been excluded from our analysis. This highlights a complication in investigating complex traits and the biological complexity of homeostatic mechanisms: it is hard to encompass the variability in a given lipid trait while keeping the genetic risk score specific; this is likely reflected by the magnitude of the *r* ^2^ statistics in our score validation (Table 1). We would have liked to adjust for statin use in our analyses; however data were not available. By including patients who are taking statins in our analysis we may have reduced the true effects of the genetic risk scores on prostate cancer outcomes.

We conclude that this study presents tentative evidence of a potential role for LDL and TG levels in prostate cancer etiology, but not for HDL. These inferences assume Mendelian randomization assumptions (such as absence of pleiotropy) hold. Future work will involve developing stronger and more specific instruments for circulating lipid traits, and making use of new instrumental variable methods, such as Egger regression which controls for pleiotropy 37. If confirmed, these findings are potentially of translational importance as they indicate that lowering LDL cholesterol may be beneficial in reducing risk of high‐grade prostate cancer.

## Conflict of Interest

None declared.

## Supporting information


**Table S1.** Genotyped versus imputed SNPs.
**Table S2.** SNPs used to generate allele scores for an unfavorable lipid profile.
**Figure S1.** Meta‐analysis OR prostate cancer per unit increase in genetic risk score (SD trait).
