# Supplementary material for: Blood lipids and prostate cancer: a Mendelian randomization analysis
Source: Cancer Med. 2016 Mar 19;5(6):1125–36. doi: 10.1002/cam4.695 (PMC4924371; doi:10.1002/cam4.695)
Supplement: Supplementary file 1 — Table S1. Genotyped versus imputed SNPs. Table S2. SNPs used to generate allele scores for an unfavorable lipid profile. Figure S1. Meta‐analysis OR prostate cancer per unit increase in genetic risk score (SD trait). [file CAM4-5-1125-s001.docx]

**Supplementary table 1:** Genotyped vs imputed SNPs

| **SNP** | **Risk allele F** | | **r^2^ imputed** | **All prostate cancer**  **OR (95% CI), p value** | |
| --- | --- | --- | --- | --- | --- |
|  | **genotyped** | **imputed** |  | **genotyped** | **imputed** |
| rs1129555  (n=41,939) | 0.29 | 0.29 | 1 | 1.03 (0.99, 1.07), 0.22 | 1.03 (0.99, 1.07), 0.22 |
| rs2814944  (n=41,943) | 0.16 | 0.16 | 1 | 1.01 (0.96, 1.05), 0.85 | 1.01 (0.96, 1.05), 0.85 |
| rs1084651  (n=41,936) | 0.18 | 0.18 | 1 | 1.03 (1.00, 1.07), 0.09 | 1.03 (1.00, 1.07), 0.09 |
| rs1042034  (n=41,932) | 0.21 | 0.21 | 1 | 1.02 (0.98, 1.05), 0.36 | 1.02 (0.98, 1.05), 0.36 |
| rs10195252  (n=41,942) | 0.59 | 0.59 | 1 | 1.00 (0.97, 1.03), 0.88 | 1.00 (0.97, 1.03), 0.88 |

| **Gene**  **Supplementary table 2:** SNPs used to generate allele scores for an unfavourable lipid profile | **SNP** | **Chromosome: position^γ^** | **Associated trait** | **Minor/major allele** | **Risk allele^†^** | **CEU Risk allele** | **Risk allele frequency in PRACTICAL controls** | ***P* value for the SNP effect in the referenced GWAS** | **Paper Ref** | **Effect size (SD trait)*** |
| --- | --- | --- | --- | --- | --- | --- | --- | --- | --- | --- |
| APOH-PRXCA | rs1801689 | 17:64210580 | LDL | C/A | C | 0.031 | 0.040 | 1x10^-11^ | [1] | 0.103 |
| MTMR3 | rs5763662 | 22:30378703 | LDL | T/C | T | 0.025 | 0.023 | 1.2x10^-08^ | [1] | 0.077 |
| ABO | rs649129 | 9:136154304 | LDL | T/C | T | 0.217 | 0.216 | 7.85x10^-22^ | [2] | 0.053 |
| ANXA9-CERS2 | rs267733 | 1:150958836 | LDL | G/A | A | 0.872 | 0.842 | 5.3x10^-09^ | [1] | 0.033 |
| FADS1–2–3 | rs174583 | 11:61609750 | LDL | T/C | C | 0.650 | 0.644 | 1.17x10^21^ | [2] | 0.030 |
| GPAM | rs1129555 | 10:113910721 | LDL | A/G | A | 0.283 | 0.289 | 2.14x10^-09^ | [2] | 0.028 |
| MAFB | rs2902941 | 20:39091487 | LDL | G/A | A | 0.688 | 0.664 | 1.11x10^-08^ | [2] | 0.025 |
| EHBP1 | rs2710642 | 2:63149557 | LDL | G/A | A | 0.659 | 0.672 | 6.1x10^-09^ | [1] | 0.024 |
| FN1 | rs1250229 | 2:216304384 | LDL | T/C | C | 0.758 | 0.748 | 3.1x10^-08^ | [1] | 0.024 |
| BRCA2 | rs4942486 | 13:32953388 | LDL | C/T | T | 0.540 | 0.475 | 2x10^-11^ | [1] | 0.024 |
| OSBPL7 | rs7225700 | 17:45391804 | LDL | T/C | C | 0.603 | 0.653 | 3.92x10^-09^ | [2] | 0.022 |
| LCAT | rs16942887 | 16:67928042 | HDL | A/G | G | 0.907 | 0.876 | 8.39x10^-33^ | [3] | 0.080 |
| APOB | rs1042034 | 2:21225281 | HDL | C/T | C | 0.195 | 0.214 | 1.36x10^-45^ | [2] | 0.057 |
| SLC39A8 | rs13107325 | 4:103188709 | HDL | T/C | T | 0.097 | 0.075 | 7.2x10^-11^ | [3] | 0.053 |
| LRP4 | rs3136441 | 11:46743247 | HDL | C/T | T | 0.964 | 0.855 | 3.48x10^-18^ | [3] | 0.049 |
| FADS1–2–3 | rs174601 | 11:61623140 | HDL | T/C | T | 0.383 | 0.381 | 1.5x10^-22^ | [2] | 0.046 |
| TTC39B | rs643531 | 9:15296034 | HDL | C/A | C | 0.146 | 0.131 | 1.3x10^-13^ | [2] | 0.046 |
| COBLL1 | rs12328675 | 2:165540800 | HDL | C/T | T | 0.853 | 0.875 | 2x10^-15^ | [3] | 0.043 |
| SCARB1 | rs838880 | 12:125261593 | HDL | C/T | T | 0.712 | 0.662 | 2.58x10^-14^ | [3] | 0.039 |
| TRIB1 | rs10808546 | 8:126495818 | HDL | T/C | C | 0.589 | 0.597 | 6.35x10^-19^ | [2] | 0.039 |
| TMEM176A | rs17173637 | 7:150529449 | HDL | C/T | C | 0.058 | 0.101 | 2x10^-08^ | [1] | 0.036 |
| LPA | rs1084651 | 6:161089817 | HDL | A/G | A | 0.120 | 0.174 | 2.97x10^-08^ | [2] | 0.035 |
| OR4C46 | rs11246602 | 11:51512090 | HDL | C/T | T | 0.863 | 0.865 | 2x10^-10^ | [1] | 0.034 |
| ARL15 | rs6450176 | 5:53298025 | HDL | A/G | A | 0.261 | 0.258 | 4.98x10^-08^ | [4] | 0.031 |
| C6orf106 | rs2814944 | 6:34552797 | HDL | A/G | A | 0.143 | 0.163 | 3.81x10^-09^ | [2] | 0.031 |
| SETD2 | rs2290547 | 3:47061183 | HDL | A/G | A | 0.200 | 0.183 | 4x10^-09^ | [1] | 0.030 |
| UBE2L3 | rs181362 | 22:21932068 | HDL | T/C | T | 0.168 | 0.216 | 1.11x10^-08^ | [3] | 0.029 |
| STAB1 | rs13326165 | 3:52532118 | HDL | A/G | G | 0.792 | 0.802 | 9x10^-11^ | [1] | 0.029 |
| HAS1 | rs17695224 | 19:52324216 | HDL | A/G | A | 0.239 | 0.253 | 2x10^-13^ | [1] | 0.029 |
| CMIP | rs2925979 | 16:81534790 | HDL | T/C | T | 0.305 | 0.316 | 2.09x10^-11^ | [3] | 0.028 |
| TRPS1 | rs2293889 | 8:11659919 | HDL | T/G | T | 0.420 | 0.409 | 5.77x10^-11^ | [3] | 0.028 |
| MVK | rs7134594 | 12:110000193 | HDL | C/T | C | 0.487 | 0.478 | 6.88x10^-15^ | [3] | 0.028 |
| MC4R | rs12967135 | 18:57849023 | HDL | A/G | A | 0.263 | 0.230 | 6.58x10^-09^ | [3] | 0.027 |
| AMPD3 | rs2923084 | 11:10388782 | HDL | G/A | G | 0.155 | 0.201 | 4.62x10^-08^ | [3] | 0.026 |
| PDE3A | rs7134375 | 12:20473758 | HDL | A/C | C | 0.606 | 0.578 | 3.48x10^-08^ | [3] | 0.026 |
| MOGAT2-DGAT2 | rs499974 | 11:75455021 | HDL | A/C | A | 0.195 | 0.181 | 1x10^-08^ | [1] | 0.026 |
| CITED2 | rs605066 | 6:139829666 | HDL | C/T | C | 0.446 | 0.425 | 2.55x10^-08^ | [3] | 0.025 |
| LACTB | rs2652834 | 15: 63396867 | HDL | A/G | A | 0.217 | 0.204 | 8.75x10^-09^ | [3] | 0.025 |
| ATG7 | rs2606736 | 3:11400249 | HDL | C/T | T | 0.594 | 0.618 | 5x10^-08^ | [1] | 0.025 |
| RBM5 | rs2013208 | 3:50129399 | HDL | T/C | C | 0.540 | 0.503 | 9x10^-12^ | [1] | 0.025 |
| FAM13A | rs3822072 | 4:89741269 | HDL | A/G | A | 0.455 | 0.465 | 4x10^-12^ | [1] | 0.025 |
| KAT5 | rs12801636 | 11:65391317 | HDL | A/G | G | 0.783 | 0.780 | 3x10^-08^ | [1] | 0.024 |
| ANGPTL1 | rs4650994 | 1:178515312 | HDL | G/A | A | 0.571 | 0.513 | 7x10^-09^ | [1] | 0.021 |
| HDGF-PMVK | rs12145743 | 1:156700651 | HDL | G/T | T | 0.681 | 0.658 | 2x10^-08^ | [1] | 0.020 |
| GSK3B | rs6805251 | 3:119560606 | HDL | T/C | C | 0.668 | 0.607 | 1x10^-08^ | [1] | 0.020 |
| ZBTB42-AKT1 | rs4983559 | 14:105277209 | HDL | G/A | A | 0.624 | 0.607 | 1x10^-08^ | [1] | 0.020 |
| ADH5 | rs2602836 | 4:100014805 | HDL | A/G | G | 0.568 | 0.575 | 5x10^-08^ | [1] | 0.019 |
| FRMD5 | rs2929282 | 15:44245931 | TG | T/A | T | 0.000 | 0.046 | 1.63x10^-11^ | [3] | 0.056 |
| PLTP | rs4810479 | 20:44545048 | TG | C/T | C | 0.243 | 0.249 | 4.69x10^-18^ | [2] | 0.036 |
| AKR1C4 | rs1832007 | 10:5254847 | TG | G/A | A | 0.842 | 0.846 | 2x10^-12^ | [1] | 0.033 |
| NAT2 | rs1495743 | 8:18273300 | TG | G/C | G | 0.288 | 0.230 | 4.11x10^-14^ | [2] | 0.032 |
| MAP3K1 | rs9686661 | 5:55861786 | TG | T/C | T | 0.195 | 0.172 | 1.32x10^-10^ | [3] | 0.028 |
| JMJD1C | rs10761731 | 10:65027610 | TG | T/A | A | 0.536 | 0.585 | 3.48x10^-12^ | [3] | 0.026 |
| CYP26A1 | rs2068888 | 10:94839642 | TG | A/G | G | 0.513 | 0.552 | 2.38x10^-08^ | [3] | 0.025 |
| MPP3 | rs8077889 | 17:41878166 | TG | C/A | C | 0.235 | 0.209 | 1x10^-08^ | [1] | 0.025 |
| KLHL8 | rs442177 | 4:88030261 | TG | G/T | T | 0.642 | 0.581 | 8.65x10^-12^ | [3] | 0.024 |
| MSL2L1 | rs645040 | 3:135926622 | TG | G/T | T | 0.770 | 0.782 | 2.52x10^-08^ | [3] | 0.024 |
| CTF1 | rs11649653 | 16:30918487 | TG | G/C | C | 0.600 | 0.601 | 3.35x10^-08^ | [3] | 0.023 |
| PINX1 | rs11776767 | 8:10683929 | TG | C/G | C | 0.405 | 0.362 | 1.3x10^-08^ | [3] | 0.022 |
| COBLL1 | rs10195252 | 2:165513091 | TG | C/T | T | 0.558 | 0.590 | 1.63x10^-10^ | [2] | 0.022 |
| MET | rs38855 | 7:116358044 | TG | A/G | A | 0.486 | 0.533 | 2x10^-08^ | [1] | 0.019 |
| PLA2G6 | rs5756931 | 22:38546033 | TG | C/T | T | 0.644 | 0.614 | 3.82x10^-08^ | [3] | 0.017 |

^†^Associated with unfavourable lipid profile (increased LDL /TG or decreased HDL).

^γ^Position based on Genome Reference Consortium Human Build 37 patch release 10.

*Effect sizes are standard deviation changes in trait per risk allele (38.99mg/dL, 15.82mg/dL and 91.99mg/dL for LDL, HDL and TG respectively, calculated based on data from the Global lipids genetics consortium)

Highlighted SNPs were genotyped as well as imputed in PRACTICAL

[1]: Global Lipids Genetics C, Willer CJ, Schmidt EM, et al. Discovery and refinement of loci associated with lipid levels. *Nature genetics* 2013; **45**(11): 1274-83

[2]: Isaacs A, Willems SM, Bos D, et al. Risk scores of common genetic variants for lipid levels influence atherosclerosis and incident coronary heart disease. *Arteriosclerosis, thrombosis, and vascular biology* 2013; **33**(9): 2233-9

[3]: Teslovich TM, Musunuru K, Smith AV, et al. Biological, clinical and population relevance of 95 loci for blood lipids. *Nature* 2010; **466**(7307): 707-13.

**
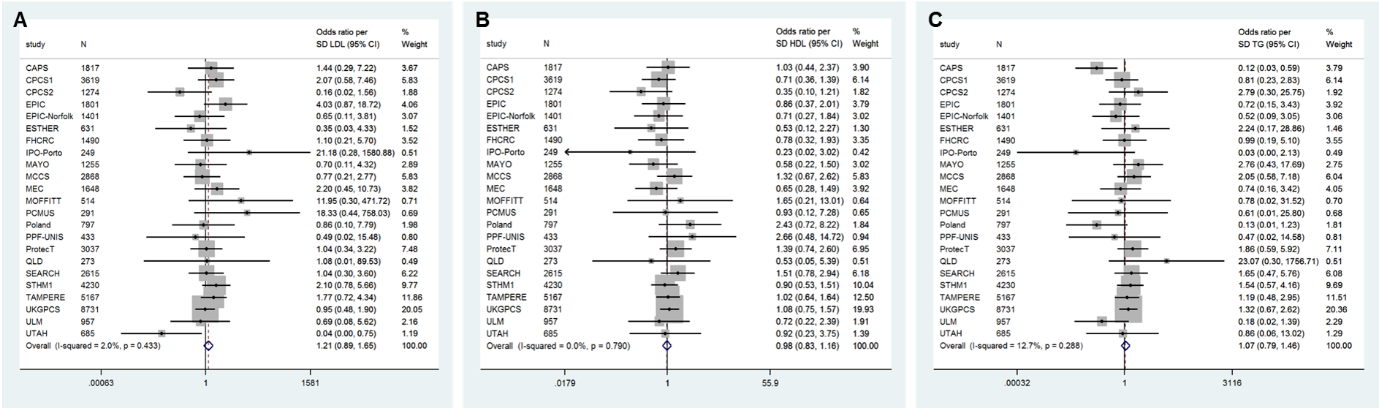
**

**Supplementary figure 1**: Meta analysis OR prostate cancer per unit increase in genetic risk score (SD trait). (**A**) LDL (low density lipoprotein): OR 1.21 (95% CI; 0.89, 1.65) p=0.22, 22,733 cases, 23,050 controls. (**B**) HDL (high density lipoprotein): OR 0.98 (95% CI; 0.83, 1.16), p=0.80, 22,733 cases, 23,050 controls. (**C**) TG (triglyceride): OR 1.07 (95% CI; 0.79, 1.46), p=0.67 22,733 cases, 23,050 controls. Adjusted for top 8 principle components (including EPIC Norfolk).
